# Perception and Regulatory Principles of Microbial Growth Control

**DOI:** 10.1371/journal.pone.0126244

**Published:** 2015-05-20

**Authors:** Armin S. Khonsari, Markus Kollmann

**Affiliations:** Mathematische Modellierung biologischer Systeme, Heinrich-Heine-Universität, Düsseldorf, Germany; University of Crete, GREECE

## Abstract

Fast growth represents an effective strategy for microbial organisms to survive in competitive environments. To accomplish this task, cells must adapt their metabolism to changing nutrient conditions in a way that maximizes their growth rate. However, the regulation of the growth related metabolic pathways can be fundamentally different among microbes. We therefore asked whether growth control by perception of the cell’s intracellular metabolic state can give rise to higher growth than by direct perception of extracellular nutrient availability. To answer this question, we created a simplified dynamical computer model of a cellular metabolic network whose regulation was inferred by an optimization approach. We used this model for a competing species experiment, where a species with extracellular nutrient perception competes against one with intracellular nutrient perception by evaluating their respective average growth rate. We found that the intracellular perception is advantageous under situations where the up and down regulation of pathways cannot follow the fast changing nutrient availability in the environment. In this case, optimal regulation ignores any other nutrients except the most preferential ones, in agreement with the phenomenon of catabolite repression in prokaryotes. The corresponding metabolic pathways remain activated, despite environmental fluctuations. Therefore, the cell can take up preferential nutrients as soon as they are available without any prior regulation. As a result species that rely on intracellular perception gain a relevant fitness advantage in fluctuating nutrient environments, which enables survival by outgrowing competitors.

## Introduction

One of the most essential aspects of living cells is growth and its associated control to fit the organisms’ needs. In human, selection for fast and selfish growth can result in cancer, while it represents a very effective evolutionary strategy for microorganisms to survive in a competitive environment. The reproductive success of microbial organism depends on the fast and precise adjustment of their growth rate to the actual environmental condition [1]. The reason is that most microbes live in a highly competitive environment where fast and effective transfer of available nutrients into biomass can give a significant fitness advantage [2].

Selection for fast growth leads to phenomena such as overflow metabolism [3–5], where fast but wasteful conversion of glucose into biomass can be of advantage in comparison to the effective use of nutrients. The overflow metabolism of *E.coli* is also known as Crabtree effect in *S. cerevisiae* and as Warburg effect in cancer cells [6]. Another regulatory phenomena that is associated with fast growth and is commonly used among many bacteria and other microbes is carbon catabolite repression (CCR) [7–9]. To grow fast microbes selectively utilize preferred carbon sources in a hierarchical manner. In the presence of a preferred sugar such as glucose, CCR causes metabolic enzymes of alternative carbon sources to be expressed at low rate and can additionally reduce their activity.

There is strong evidence that growth dependent phenomena such as overflow metabolism or CCR are the consequence of a metabolic regulation or growth control in response to extracellular nutrient availability. Further, it seems possible that the perception of extracellular nutrient availability plays an important role in growth control [10], as it is the primary information cellular response is based on. We define two distinct types of perception, termed intracellular and extracellular perception. In the case of extracellular perception the cell regulates its metabolism exclusively in response to extracellular nutrient information, while in the case of intracellular perception microbes indirectly recognize nutrient availability by perceiving the intracellular metabolic state. The intracellular perception is motivated by experimental observations [11–13] of microbes, e.g. *E.coli*, which do not possess any extracellular carbohydrate receptors, like the Rgt2 and Snf3 glucose sensors of yeast [14, 15]. These microbes should be capable of perceiving extracellular nutrient availability indirectly from intracellular metabolic states. Intuitively, the extracellular perception should lead to a more precise and fast adaptation to nutrient availability, since changes in the environment can be perceived faster and to higher accuracy. Here, the question arises whether exclusive intracellular perception can result in a growth benefit in presence of fast fluctuating nutrient concentrations. Following this question, we are interested in which frequency regimes the exclusive perception of intracellular nutrient concentration is evolutionary more beneficial than the exclusive perception of extracellular nutrient concentrations. Furthermore, what are the regulatory principles causing this benefit in average growth rate or fitness and can the regulatory phenomenon of carbon catabolite repression be understood by means of nutrient perception?

To give an answer to these questions and an explanation how the integration of the perception strategies for growth control contribute to shape growth rate in microorganisms, we will introduce a simplified replicator model for microbial growth. The replicator model consists of a minimal metabolic network, ribosomes, and a controller that can detect intracellular and extracellular metabolite concentrations. Optimal growth control is realized by minimizing the difference between the actual intracellular concentrations of metabolites and precursors and their desired concentrations, which is determined by the perceived nutrient availability. Using this simplified model we are able to show that growth control by perception of extracellular nutrient concentrations is of selective advantage if environmental conditions change slowly over time. If environmental conditions change fast in comparison to the minimum generation time, gene regulation and protein turnover will lag behind and the model predicts that in this case sensing the intracellular precursor state is of advantage.

## Methods

### Self-replicator model

**Fig 1:**
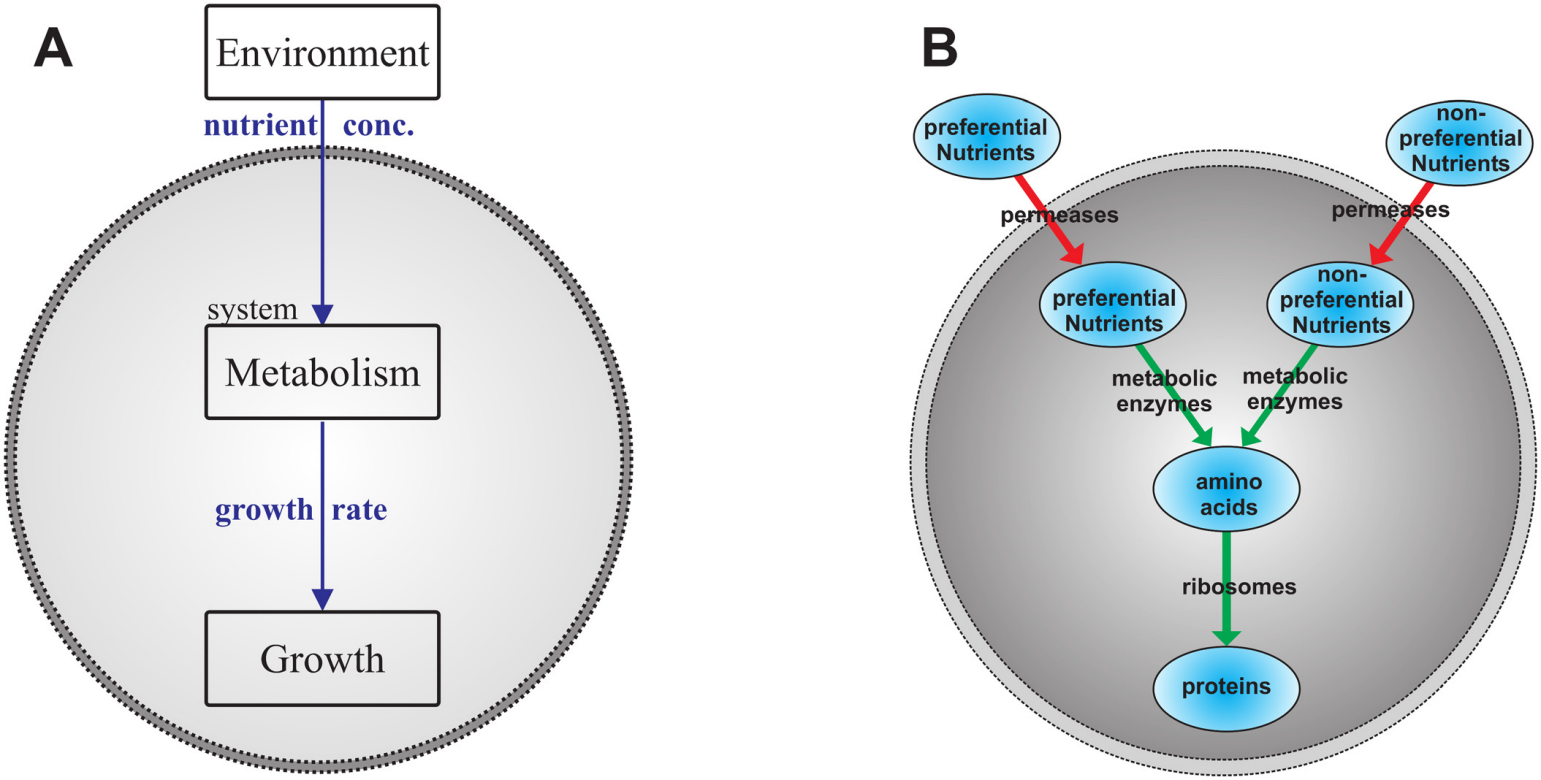
The metabolism of the self-replicator shown in two possible representation. (A) Block diagram: blocks symbolize processes and arrows associated inputs and outputs. The big dashed circle distinguishes between intracellular and extracellular processes. The process of growth is caused by the underlying metabolism which in turn depends on the nutrient availability in the environment. (B) Pool diagram: ellipses represent the protein and metabolite pools. Red arrows symbolize uptake transports and green arrows stand for metabolic pathway fluxes. The self-replicator consists of two metabolic pathways—one for preferential nutrients and one for non-preferential ones.

The first step in modeling a system is to understand the main features which have a relevant effect on the studied phenomenon or scientific objective. These features are taken to construct the most simple model which still suffices to reproduce reality. In this study we are interested in fast growing unicellular organisms in changing environments with focus on cell metabolism and its regulation. Growth is a consequence of the underlying metabolic fluxes and growth rate is affected by changes in metabolic rate which in turn can be a result of environmental changes (see Fig 1A). In the following, we define growth by the amount of protein that is synthesized. Focusing exclusively on the protein content and thereby neglecting other cellular components is legitimate since the protein synthesis capacity of a cell remains approximately constant over time [16, 17].

The next question is how a real-life metabolism can be further simplified and generalized, to avoid inclusion of too many molecular details. Molenaar et al. [2] have successfully shown that simple self-replicating systems (self-replicators) qualitatively reproduce the regulation of major cellular components (protein, lipids, etc.) for unicellular organisms. The simplest self-replicator consists of ribosomes which synthesize themselves by means of precursors (real-life example [18]). In this work we rely on a slightly more complex architecture which is obtained by adding transporters and metabolic pathways to the simple self-replicator model.

The whole self-replicating system, as sketched in Fig 1B, consists of a metabolic flux network, where metabolite pools are connected by biochemical reactions catalyzed by specific enzymes. For the sake of simplicity and without loss of generality, it is assumed that there are only two types of time varying nutrient components, namely a preferential sugar (PS) and a non-preferential sugar (NPS), which both can be growth limiting. All other compounds that are required for growth are assumed to be available in excess. Further, we assume that the self-replicating system will be situated in a surrounding that periodically switches between a PS and an NPS environment. As only two nutrient components change over time, our simplified cell comprises two catabolic pathways. The external nutrients can be imported into the cell by specific permeases, where they are transformed into metabolic precursors, i.e. amino acids, as the only precursor in the system. Using amino acids, ribosomes synthesize the five distinct enzyme types that the self-replicator consists of, including themselves. These five enzymes constitute the total amount of proteins belonging to one self-replicator. Their relative share of the total protein amount influences the protein synthesis rate, i.e. growth rate.

Each metabolic pathway, represented by the fluxes and arrows in Fig 1B, can be thought to be catalyzed by a group of enzymes with concentrations ${\hat{E}}_{i}$. The effective enzyme concentration of one whole pathway *j* is expressed as $E_{j}=\sum_{i}{\hat{E}}_{i}$. It is assumed that the maximum concentration of the proteome does not exceed a constant proteome density *E* ^max^ = ∑_*j*_ *E*_*j*_ [19, 20]. The overall protein mass density of the whole population is defined as total protein mass *M* ^tot^ of the population per total cell volume of the population *V* ^pop^.

$$\begin{array}{c} E^{\text{max}}:=\frac{M^{\text{tot}}\left(t\right)}{V^{\text{pop}}\left(t\right)}=\text{const.} \end{array}$$ (1)

Note that *M* ^tot^ and *V* ^pop^ are quantities that are measured in batch culture experiments and that *E* ^max^ corresponds to the population averaged cellular protein concentration. In what follows, we assume that fast growing organisms are optimized for biomass production, an assumption which is strongly supported by recent experimental results [11, 16]. In order to describe the system dynamics with the necessary accuracy, we introduce a mathematical description for the metabolite and enzyme pool dynamics.

### Metabolite pool dynamics

A metabolite pool is characterized by its mass density. The mass density [*X*] of metabolite *X* is denoted as metabolite mass *m*_*X*_(*t*) per population volume *V* ^pop^.

$$\begin{array}{c} \left[X\right]\left(t\right):=\frac{m_{X}\left(t\right)}{V^{\text{pop}}\left(t\right)} \end{array}$$ (2)

**Fig 2:**
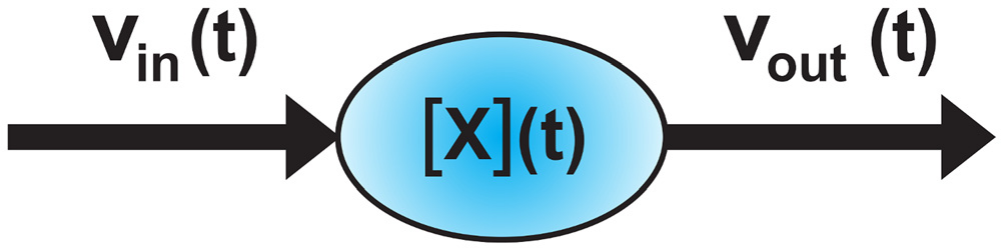
Concentration dynamic of arbitrary metabolite X. While the outflow rate *v*_out_(*t*) depends on the metabolite pool concentration [*X*](*t*), the inflow rate *v*_in_(*t*) is independent of [*X*](*t*) and is subject to an upstream pool.

Alternatively, one can use the particle density $\tilde{\left[X\right]}:=n_{X}/V^{\text{pop}}$, which is the amount of particle in *mol* over population volume in *l*. (This definition is utilized for flux balance analysis with the Matlab toolbox *cobra* [21].) All metabolite pool dynamics are defined by continuity equations. Furthermore the concentration and fluxes must always be positive, as it is hinted in Fig 2.

- Continuity: $\frac{d[X](t)}{dt}=v_{\text{in}}(t)-v_{\text{out}}(t)$
- Positivity: [*X*](*t*) ≥ 0 and *v*_*i*_ ≥ 0

The outflow rate *v*_out_(*t*) depends on the pool concentration [*X*](*t*) in conjunction with the related enzyme concentration *E*_*X*_, whereas there is no direct dependency to the inflow rate *v*_in_(*t*). Due to the existence of a single metabolic network, all pools are connected. This gives rise to interpret all inflow rates as an outflow rate of an upper pool [*Y*](*t*). Hence for a linear pathway, it is sufficient to define the outflow rate as:

$$\begin{array}{c} v_{X}\left(t\right):=v_{out}\left(t\right)=\frac{\left[X](t\right)}{K_{M}^{X}+\left[X\right]\left(t\right)}\cdot k_{X}\cdot E_{X}\left(t\right)\;, \end{array}$$ (3)

where $K_{M}^{X}$ is the Michaelis-Menten constant and *k*_*X*_ is the catalytic rate of the enzyme reaction. The inflow rate has the same expression as above with the only difference of being defined by the upper pool [*Y*](*t*), i.e. *v*_in_ = *v*_Y_. In order to work with normalized quantities, the relative mass *λ*_*X*_ of metabolite *X* is introduced by

$$\begin{array}{c} \lambda_{X}\left(t\right):=\frac{m_{X}\left(t\right)}{M^{\text{tot}}\left(t\right)}=\frac{\left[X](t\right)}{E^{\text{max}}}\;. \end{array}$$ (4)

The pool dynamics follow from the defined fluxes, the continuity equation, and the definition of the relative metabolite mass.

$$\begin{array}{c} \frac{d}{dt}\lambda_{X}\left(t\right)=\frac{1}{E^{\text{max}}}\cdot \frac{d}{dt}\left[X\right]\left(t\right)=\frac{v_{Y}\left(t\right)-v_{X}\left(t\right)}{E^{\text{max}}} \end{array}$$ (5)

### Enzyme pool dynamics: regulation and growth

A mathematical description of growth control can be obtained by determining the time dynamics of growth rate and the enzyme pools. Optimal growth control is achieved by regulating metabolic fluxes in a way that maximizes growth rate. The metabolic fluxes are driven by their related enzyme concentrations and extra- and intracellular metabolite concentrations. Since the latter is a not influenceable environmental factor, growth control exclusively means regulating enzyme concentrations. The optimal timing, by which this regulation is performed, is influenced by the growth rate. The reason is that the enzyme concentrations can be diluted or over-expressed due to growth. In the following, the proper quantitative definitions of growth, growth rate and regulation will be developed in order to obtain the basis for deriving their time dynamics.

**Definition** To describe **cellular growth**, the protein mass is a better quantity than the corresponding concentration. The time evolution of the total protein mass *M* ^tot^(*t*) is proportional to its cell population volume *V* ^pop^(*t*), since we assume a constant total protein concentration *E* ^max^. Consequently, *d*_*t*_ *E* ^max^ = *d*_*t*_(*M* ^tot^(*t*)/*V*_cell_(*t*)) = 0, despite of increasing mass and volume. Hence, the total protein mass and the associated total protein mass flux are the appropriate quantities for describing cell growth and growth rate, respectively.

To describe the **regulatory dynamics** of the various enzyme pools we introduce the relative enzyme mass *ϕ*_*j*_ = *M*_*j*_/*M*_tot_ by the ratio of the enzyme mass *M*_*j*_(*t*) of a metabolic pathway *j* to the total protein mass *M* ^tot^(*t*). As the cellular system tends to maximize its growth rate, which is represented by the synthesized protein mass per time unit, optimal growth rate is a result of an optimized metabolism. In this model, the only way of tuning metabolism is by means of redistributing the enzyme concentrations *E*_*j*_(*t*) of metabolic pathways. This is due to a constant intracellular protein concentration, which is maintained by the cell to ensure efficiency of central cellular processes, such as protein folding [19, 22]. In analogy to the relative enzyme mass *ϕ*_*j*_, one can define a relative enzyme concentration *E*_*j*_(*t*)/*E* ^max^, which can be shown to be related:

$$\begin{array}{c} \Phi_{j}\left(t\right):=\frac{M_{j}\left(t\right)}{M_{\text{tot}}\left(t\right)}=\frac{\left(M_{j}\left(t\right)/V^{\text{pop}}\left(t\right)\right)}{\left(M^{\text{tot}}\left(t\right)/V^{\text{pop}}\left(t\right)\right)}=\frac{E_{j}\left(t\right)}{E^{\text{max}}}\;, \end{array}$$ (6)

where *M* ^tot^(*t*) = ∑_*j*_ *M*_*j*_(*t*) and ∑_*j*_ *ϕ*_*j*_(*t*) = *ϕ* ^max^ = 1. Both quantities can likewise be used to describe metabolic regulation. But the relative enzyme mass *ϕ*_*j*_(*t*) is more favorable, because it stands in direct relation to the definition of cellular growth, and will be used for the derivation of the regulatory dynamics below.

**Regulation** The regulatory dynamics are obtained by taking the time derivative of the relative protein mass *ϕ*_*j*_(*t*). The time derivative *d*/*dtϕ*_*j*_(*t*) depends on the derivatives of the total protein mass *d*_*t*_ *M* ^tot^(*t*) and the pathway protein mass *d*_*t*_ *M*_*j*_(*t*). For this purpose one can define the following useful relation between both mass quantities:

$$\begin{array}{c} \gamma_{j}\left(t\right):=\frac{\frac{d}{dt}M_{j}\left(t\right)}{\frac{d}{dt}M^{\text{tot}}\left(t\right)}\;, \end{array}$$ (7)

where ∑_*j*_ *γ*_*j*_(*t*) ≡ 1. The *relative synthesis rate* *γ*_*j*_(*t*) is the synthesis rate of enzymes from pathway *j* with respect to the overall synthesis rate. It can be interpreted as the fraction of protein synthesis capacity that is assigned to enzyme *j*. This synthesis capacity can be generalized to other biological regulatory mechanisms, like the amount of mRNA, tRNA etc. Deriving the relative protein mass and using relation Eq (7) yields the ordinary differential equation for the regulatory dynamics.

$$\begin{array}{ccc} \frac{d}{dt}(\frac{M_{j}\left(t\right)}{M^{\text{tot}}\left(t\right)}) & = & \frac{M^{\text{tot}}\left(t\right)\cdot \left(d_{t}M_{j}\left(t\right)\right)-M_{j}\left(t\right)\cdot \left(d_{t}M^{\text{tot}}\left(t\right)\right)}{{\left(M^{\text{tot}}\left(t\right)\right)}^{2}}\\ & = & \frac{\frac{d}{dt}M^{\text{tot}}\left(t\right)}{M^{\text{tot}}\left(t\right)}\cdot (\frac{\frac{d}{dt}M_{j}\left(t\right)}{\frac{d}{dt}M^{\text{tot}}\left(t\right)}-\frac{M_{j}\left(t\right)}{M^{\text{tot}}\left(t\right)})\\ \frac{d}{dt}\Phi_{j}\left(t\right) & = & \frac{\frac{d}{dt}M^{\text{tot}}\left(t\right)}{M^{\text{tot}}\left(t\right)}\cdot [\gamma_{j}\left(t\right)-\Phi_{j}\left(t\right)] \end{array}$$ (8)

The differential equation (Eq (8)) describes the change of the relative enzyme mass for each pathway. This time-dependency of enzymatic resources represents the regulatory dynamics of a single cell, under the simplifying assumptions introduced before. The relative enzyme mass *ϕ*_*j*_ tends toward the synthesis rate ratio *γ*_*j*_ with the population size independent growth rate *v*_growth_ = *d*_*t*_ *M* ^tot^/*M* ^tot^. Eq (8) describes a growing cellular system that redistributes its protein synthesis capacity in regulatory manner, under the constraint ∑_*j*_ *γ*_*j*_(*t*) ≡ 1.

There are three scenarios with respect to regulation. Using relation Eq (8), one can find following interpretation:

1. **Dilution**: enzyme concentration decreases
$\gamma_{j}(t)<\phi_{j}(t)\Leftrightarrow \frac{d}{dt}\frac{M_{j}(t)}{M^{\text{tot}}(t)}<0\Leftrightarrow \frac{d}{dt}E_{j}(t)<0$
  If the relative synthesis rate *γ*_*j*_ is smaller than the relative enzyme mass *ϕ*_*j*_, the synthesis rate of enzyme *j* will be smaller than the growth rate. Hence, a dilution effect will be initiated and relative enzyme mass and enzyme concentration *E*_*j*_ will decrease.
2. **Over-expression**: enzyme concentration increases
$\gamma_{j}(t)>\phi_{j}(t)\Leftrightarrow \frac{d}{dt}\frac{M_{j}(t)}{M^{\text{tot}}(t)}>0\Leftrightarrow \frac{d}{dt}E_{j}(t)>0$
  If the relative synthesis rate *γ*_*j*_ is larger than the relative mass *ϕ*_*j*_, enzyme *j* will be synthesized faster than the rate the cell is growing. Hence, an over-expression effect will be initiated and relative enzyme mass and enzyme concentration *E*_*j*_ will increase.
3. **Homeostasis**: enzyme concentration stays constant
$\gamma_{j}(t)=\phi_{j}(t)\Leftrightarrow \frac{d}{dt}\frac{M_{j}(t)}{M^{\text{tot}}(t)}=0\Leftrightarrow \frac{d}{dt}E_{j}(t)=0$
  If the relative synthesis rate *γ*_*j*_ is as large as the relative mass *ϕ*_*j*_, enzyme *j* will be synthesized exactly as fast as the rate the cell is growing. Hence, the cellular enzyme composition will be preserved and homeostasis is established—relative enzyme mass and enzyme concentration *E*_*j*_ will stay constant.

The system always tends to the third case, homeostasis, where following relation is established:

$$\begin{array}{c} \frac{d}{dt}M_{j}\left(t\right)=\Phi_{j}\left(t\right)\cdot \frac{d}{dt}M^{\text{tot}}\left(t\right) \end{array}$$ (9)

**Growth** In order to determine the time dependency of cellular protein mass growth, the following ordinary differential equation has to be solved:

$$\begin{array}{c} \frac{d}{dt}M^{\text{tot}}\left(t\right)=(\beta_{R}\left(t\right)\cdot k_{R}\cdot \Phi_{R}\left(t\right))\cdot M^{\text{tot}}\left(t\right)\;, \end{array}$$ (10)

where $\beta_{R}(t)=([\text{AA}])/(K_{M}^{R}+[\text{AA}])$ is the probability of amino-acid-binding to a ribosome and [AA] is the amino acid concentration. We do not consider the contribution of different amino acids because one type is sufficient for our phenomenological model, regarding previous assumptions. This differential equation, Eq (10), represents exponential growth with a time-dependent growth rate *v*_growth_(*t*): = *β*_*R*_(*t*) ⋅ *k*_*R*_ ⋅ *ϕ*_*R*_(*t*), whereas *v*_growth_(*t*) is based on Michaelis-Menten kinetics of ribosomal translation. Solving this ordinary differential equation yields the following exponential growth relation.

$$\begin{array}{c} M^{\text{tot}}\left(t\right)=M^{\text{tot}}\left(t_{0}\right)\cdot \exp(k_{R}\cdot \int_{t_{0}}^{t}\beta_{R}\left(t\right)\cdot \Phi_{R}\left(t\right)\;dt) \end{array}$$ (11)

Eq (11) can be seen as microscopic view of cellular growth, where the population’s protein mass is exponentially increased instead of the the number of cells. To transform Eq (11) into a more classical macroscopical form of cell growth, one hast to introduce the relation *M* ^tot^(*t*) = ⟨*M*_cell_⟩ ⋅ *n*(*t*), where *n*(*t*) denotes the number of cells in a population and ⟨*M*_cell_⟩ is the average proteome mass of a single cell. Applying this relation and the connection *v*_growth_(*t*) = ln2/*t*_*D*_(*t*) between growth rate *v*_growth_ and cellular doubling time *t*_*D*_ yields the macroscopic view of cellular growth.

$$\begin{array}{c} n\left(t\right)=n\left(t_{0}\right)\cdot 2^{\int_{t_{0}}^{t}\frac{1}{t_{D}\left(t\right)}\;dt} \end{array}$$ (12)

Here, the time-dependent cellular doubling time is expressed as

$$\begin{array}{c} t_{D}\left(t\right)=\frac{ln(2)}{\beta_{R}\left(t\right)\cdot k_{R}\cdot \Phi_{R}\left(t\right)}\;. \end{array}$$ (13)

Eqs (12) and (13) show that the population size doubles by a time which depends on the amino acid concentration [AA] and relative protein mass *investment* in ribosomes. The more ribosomes and amino acids are present, the shorter is the cellular doubling time and the faster is cellular growth. Assuming stable proteins, the doubling time equals the response time *t*_*R*_, i.e. the time a cell needs to respond properly to an environmental change.

### Control system

A living cell can be regarded as a control system consisting of a system to be controlled, controller, actuator, and sensors. The system to be controlled is represented by the metabolic network, while the actuator can be seen as the protein synthesis machinery, i.e. ribosomes which produce specific enzymes with a probability given by the relative protein synthesis rate *γ*_*j*_. Next, sensors and controller have to be added to the model in order to complete the control system. In the following the process of sensing will be referred to as perception and there will be only two types as will be seen below, namely intracellular perception and extracellular perception. The explicit nature of the sensors are not important for our research question, since we are only interested in the effective information content of those. The controller yields cellular regulation, which must be inferred by an mathematical optimization process.

**Fig 3:**
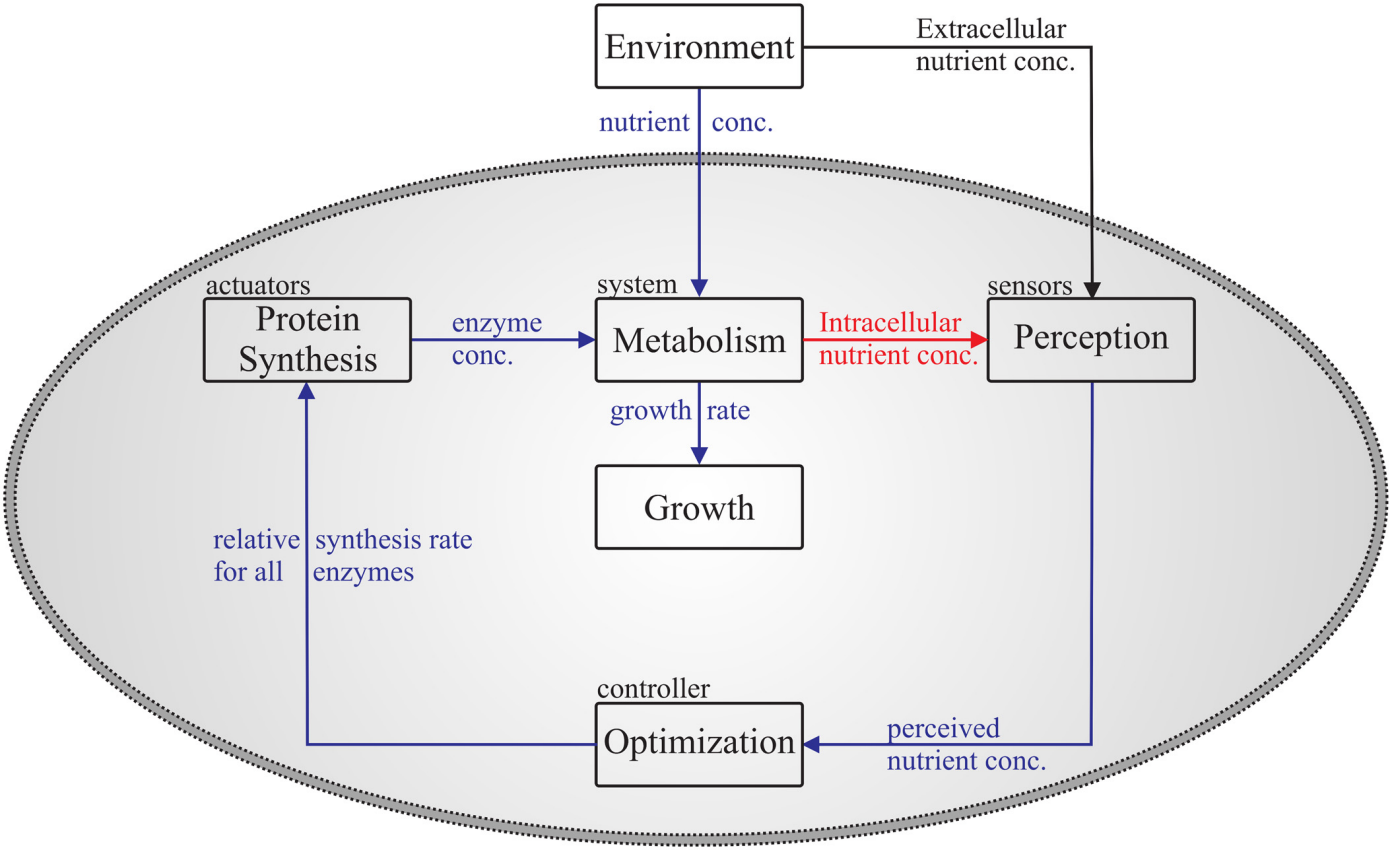
Block diagram of the whole modeled replicating system. This control system consists of a system to be controlled, namely the metabolic network, a controller, actuators and sensors for determining the metabolic pools’ relative mass. Each block represents a process, which can contain sub-processes. While blue arrows represent input and output of the different processes, the red and black arrows represent the input for intracellular and extracellular perception, respectively.

Having all pieces together, one can explain the dynamic steps of growth control by means of the control system sketched in Fig 3. The metabolic system takes up nutrients from the environment and metabolizes them into proteins, which in turn increase cell mass, i.e. the cell grows. This process is regulated by the controller which receives information about the nutrient availability from perception and hands over desired enzyme concentrations to the actuator, namely the ribosomes. The actuator implements this desired values by changing the actual enzyme concentration. This total control process is time dependent and hence explains the dynamic steps of growth control.

As mentioned, the modeled control system has a desired value and an actual value for all fluxes and enzyme concentrations. Former has to be distinguished from the optimal value. While the **desired value** is a quantity that the **actual value** aims for, the **optimal value** is a quantity which represents the global maximum or minimum of an objective function (here: growth rate). If and only if the desired value is determined under ideal conditions, it will be equal to the optimal value.

**Desired value** The desired value, which represents the control of a system, depends strongly on the information quality of the surrounding environment, namely the extracellular nutrient concentration. This information is of utmost importance for the desired value’s accuracy, that is the degree of optimality with respect to the control. Obviously, the measurement of extracellular nutrient concentration is more precise than the one of intracellular nutrient concentration. On the other hand, a highly precise determination of the actual extracellular concentration can be disadvantageous with respect to growth, in the case of a rapidly changing environment.

Another important point for the determination of the desired value is a matching amount of enzymes to their associated metabolites. This *optimal resource allocation* prohibits the waste of enzymes in the case of enzyme overproduction and prevents from a non-optimal growth rate due to the mismatch between catalytic capacity of too less enzymes and the existing larger metabolite pool [23]. A non-optimal distribution of resources will always cause a decrease in growth rate compared to the optimal state. In physical terms, optimal resource allocation is defined as the condition, in which the metabolite net inflow rate *v*_*in*_ into a pool equals the catalytic net outflow rate *v*_*out*_.

$$\begin{array}{c} \sum_{i}v_{in}^{i}\left(t\right)=\sum_{k}v_{out}^{k}\left(t\right) \end{array}$$ (14)

This assumption or condition, respectively, implicates *balanced fluxes* and constant pool concentrations for the whole network, if the environment is regarded to be constant. Therefore, it is possible to consider the pool and flux dynamics as an stationary process, where the pool concentration and flux instantaneously adapt to an new environment by tuning enzyme concentrations to the according desired values.

**Actual value** Balanced fluxes is a condition for optimality, but cannot always be achieved by the cell in reality. This is due to two major facts:

1. The information content is imprecise, e.g. because of the cell only measuring the intracellular nutrient concentration.
2. The change between different environments happens faster than the cells ability to adapt to the desired value.

Consequently, actual and desired value cannot always be identical, as it is in the case of a stationary process. It is appropriate to assume a stationary process in order to compute the desired values. But on the matter of determining the actual value, one must consider real dynamics of fluxes as well as pool concentrations.

**Defining the desired value** The desired value $\phi_{j}^{*}(\hat{t})$ at time $\hat{t}$ is defined by the relative enzyme mass *ϕ*_*j*_(*t*) which the system targets for if the environmental conditions would remain constant for $t>\hat{t}$. The proximity of the actual value to the desired value depends on how long the environment remains fixed relative to the response time *t*_*R*_ of the system. The two following limiting cases are possible, by defining *T* as the average time over which environmental conditions stay constant.

- **No adaptation (*t*_*R*_ > > *T*)**: The desired value changes at any time.
- **Total adaptation (*t*_*R*_ < < *T*)**: The desired value remains fixed until full adaptation (homeostasis).

The desired value can be defined by the stationary case of the regulatory enzyme pool dynamics in Eq (8). Above, it was assumed that optimal resource allocation or constant relative enzyme pools, respectively, is a state desired by the system. Therefore, the desired value $\phi_{j}^{*}$ follows from the condition

$$\frac{d}{dt}\Phi_{j}\left(t\right)\overset{!}{=}0$$

and corresponds to the relative enzyme mass synthesis rate *γ*_*j*_ at time *t*.

$$\begin{array}{c} \Phi_{j}^{*}\left(t\right):=\gamma_{j}\left(t\right)=\frac{\frac{d}{dt}M_{j}\left(t\right)}{\frac{d}{dt}M^{\text{tot}}\left(t\right)} \end{array}$$ (15)

The cell implements the desired value by adjusting (regulating) the synthesis rate ratio *γ*_*j*_, as Eq (15) shows. The system drives the enzyme mass ratio *ϕ*_*j*_ towards the synthesis rate ratio, regardless of the initial condition of *ϕ*(*t*_0_), i.e. pathway mass *M*_*j*_(*t*_0_) and total mass *M* ^tot^(*t*_0_).

$$\begin{array}{c} \frac{M_{j}\left(t\right)+M_{j}\left(t_{0}\right)}{M^{\text{tot}}\left(t\right)+M^{\text{tot}}\left(t_{0}\right)}\overset{t\to \infty }{⟶}\frac{\frac{d}{dt}M_{j}\left(t\right)}{\frac{d}{dt}M^{\text{tot}}\left(t\right)} \end{array}$$

In summary, the synthesis rate ratio can be regarded as the control function of the cell. By having the knowledge of the ratio *γ*_*j*_, it is possible to predict the state dynamics of the whole metabolic system. Of course, the control function has to depend on the extracellular nutrient concentrations and therefore on environmental conditions.

### Determining the optimal desired value

**Fig 4:**
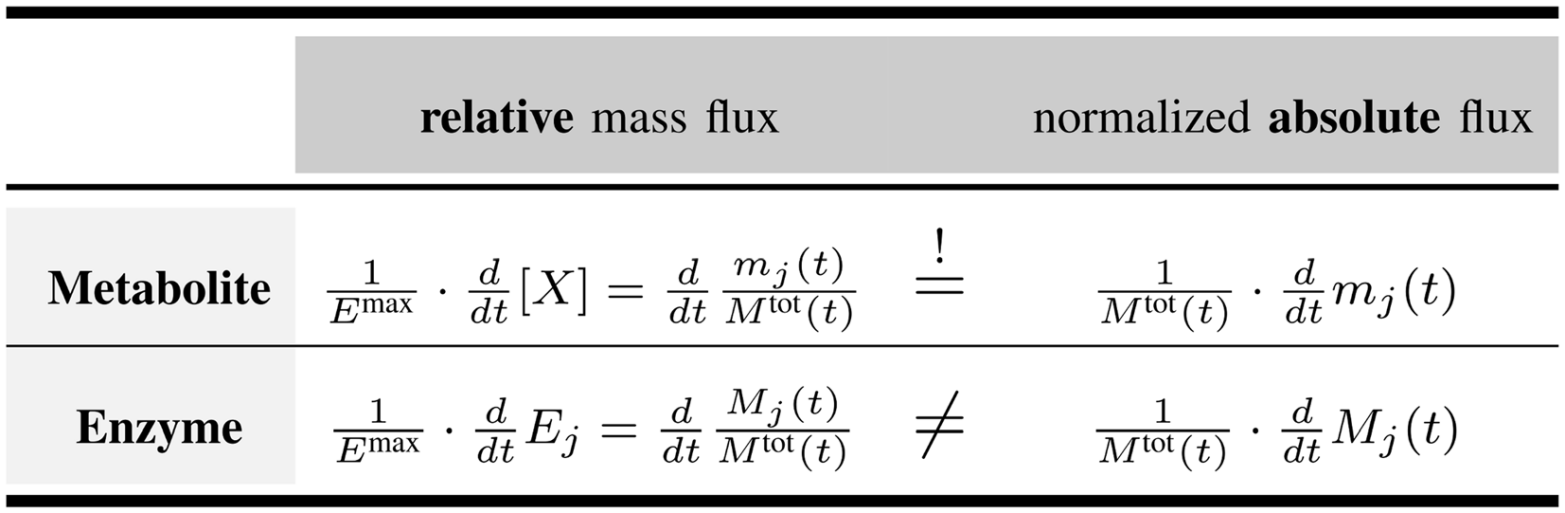
Relative mass flux and normalized absolute mass flux. Metabolic reactions happen on a much faster time scale than the rate of protein synthesis. Consequently, the relative mass flux and normalized absolute mass flux are unequal for enzymes, while they are identical for metabolites.

**Fig 5:**
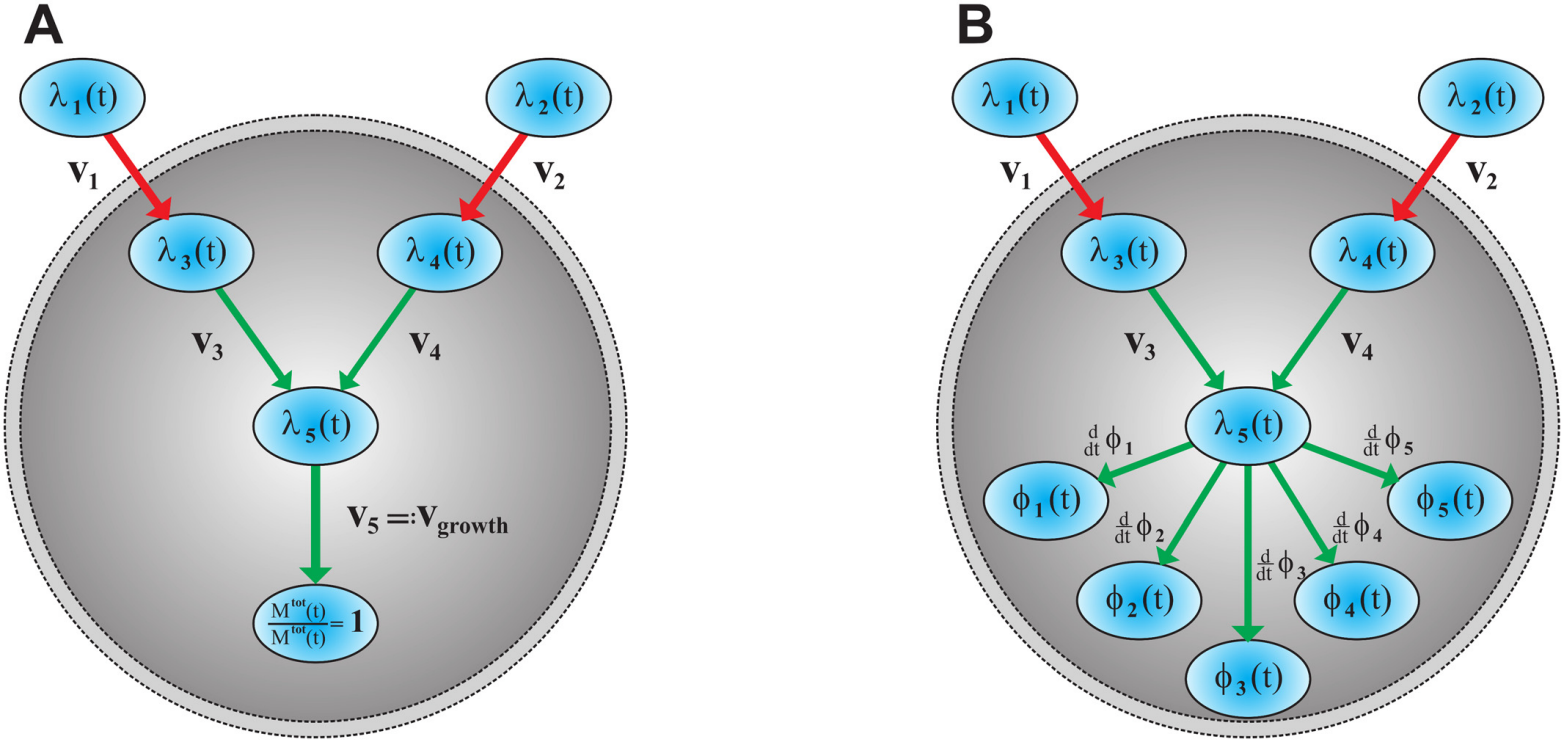
Schematic figure of the simplified metabolic network. (A) *Growth*: the arrows represent normalized absolute mass fluxes, while the metabolite and protein pools are quantified by normalized absolute mass. The growth rate *v*_5_ = *v*_growth_ is an absolute mass flux, since growth can only be understood in absolute terms. The normalization 1/*M* ^tot^ is utilized to keep quantities independent of population size. (B) *Regulation*: the arrows represent relative mass fluxes, while the metabolite and protein pools are quantified by their relative mass. The self-replicator distributes its constrained protein resources between permeases *ϕ*_1_, *ϕ*_2_, metabolic enzymes *ϕ*_3_, *ϕ*_4_, and ribosomes *ϕ*_5_. The enzyme synthesis acts as an feedback loop on the metabolic network, since metabolic fluxes *v*_*j*_ depend on enzyme levels *v*_*j*_ ∝ *ϕ*_*j*_.

**Relative and absolute mass fluxes** One has to distinguish between relative mass fluxes and normalized absolute mass fluxes, as shown in Figs 4 and 5.

$$\begin{array}{c} v_{5}=\sum_{j}\frac{\frac{d}{dt}M_{j}\left(t\right)}{M^{\text{tot}}\left(t\right)}\neq \sum_{j}\frac{d}{dt}\frac{M_{j}\left(t\right)}{M^{\text{tot}}\left(t\right)}=0 \end{array}$$

While both quantities are identical for the metabolite fluxes *v*_1_, *v*_2_, *v*_3_, *v*_4_, they are totally different for protein mass fluxes. The reason for this is the following assumption: the time dependent change of the metabolite pools happens on a much faster scale than the rate of protein synthesis. Therefore, the protein pathway mass *M*_*j*_ and the total protein mass *M* ^tot^ can be regarded as constants for the time dynamics of metabolite mass *m*_*X*_(*t*).

**Objective function and stoichiometric matrix** It is assumed that the metabolic network is optimized in such a way that the system’s growth rate is maximized [23–25]. In order to determine the desired value ϕ*(*t*) of the relative protein mass at time *t*, the metabolic network, with normalized absolute mass fluxes (*dm*_*j*_/*dt*)/*M* ^tot^ and (*dM* ^tot^/*dt*)/*M* ^tot^, has to be optimized with respect to its growth rate (see Fig 5A). This optimization has to be applied for each time *t*. The growth rate *v*_growth_, corresponding to fitness, is defined as normalized absolute protein mass flux (synthesis rate):

$$\begin{array}{c} v_{\text{growth}}:=v_{5}=\frac{\left(\frac{d}{dt}M^{\text{tot}}\left(t\right)\right)}{M^{\text{tot}}\left(t\right)}\;. \end{array}$$ (16)

Absolute fluxes are of paramount importance, since growth can only be understood in absolute terms. Normalized fluxes, specifically the normalized protein flux, are utilized because they are independent of population size.

A stoichiometric matrix *S* with three metabolites and five fluxes can be formulated for this metabolic network. As defined above, inflow fluxes are positive and outflow fluxes are negative.

$$\begin{array}{c} S:=(\begin{array}{ccccc} +1 & 0 & -1 & 0 & 0\\ 0 & +1 & 0 & -1 & 0\\ 0 & 0 & +1 & +1 & -1 \end{array}) \end{array}$$ (17)

Using this matrix, the metabolite pool dynamic can be expressed as:

$$\begin{array}{c} \frac{d}{dt}\vec{m}\left(t\right)=(\begin{array}{ccccc} +1 & 0 & -1 & 0 & 0\\ 0 & +1 & 0 & -1 & 0\\ 0 & 0 & +1 & +1 & -1 \end{array})\cdot (\begin{array}{c} v_{1}\\ v_{2}\\ v_{3}\\ v_{4}\\ v_{5} \end{array})\;, \end{array}$$ (18)

where

$$\begin{array}{c} \vec{m}\left(t\right):=(\begin{array}{c} \lambda_{3}\left(t\right)\\ \lambda_{4}\left(t\right)\\ \lambda_{5}\left(t\right) \end{array})\;. \end{array}$$ (19)

**Optimization conditions** The mathematical problem is to find the Desired values $\phi_{j}^{*}(t)$ at time *t* for a given set of actual values of the metabolite pools and extracellular nutrient concentration. The actual values of the enzyme pools are not relevant for this purpose, since the cellular system drives towards the desired value, regardless of initial conditions of the enzyme pools. Since the growth rate is a flux, the desired relative protein masses $\phi_{j}^{*}$ need to be expressed in terms of desired metabolite fluxes $v_{j}^{*}$.

$$\begin{array}{c} v_{j}^{*}\left(t\right):=\alpha_{j}^{*}\left(t\right)\cdot \Phi_{j}^{*}\left(t\right)\;, \end{array}$$ (20)

where

$$\begin{array}{c} \alpha_{j}^{*}\left(t\right):=\frac{\lambda_{j}^{*}\left(t\right)}{\frac{K_{M}^{\left(j\right)}}{E^{\text{max}}}+\lambda_{j}^{*}\left(t\right)}\cdot k_{j}\;\text{for}\;j=1,...,5\;. \end{array}$$ (21)

The relative metabolite masses λ*_j_ at time *t* represent perceived values, which can differ with respect to the type of perception and need not to be equal to the real values *λ*_*j*_(*t*). The desired relative protein masses can be obtained by maximizing the growth rate for each time *t* under following conditions.

- **Positive fluxes**: The fluxes are constrained to be positive. (By constraining the lower boundary to a non zero value, one could simulate a basal enzyme expression level, which is not done here.)
- **Optimal resource allocation**: This condition implicates constant metabolite pools and hence balanced fluxes, as can be seen by setting the time derivative of all metabolite masses to zero (see Eq (18)).
  $$\begin{array}{c} \frac{d}{dt}\vec{m}\left(t\right)\overset{!}{=}0 \end{array}$$
  $$\begin{array}{c} S\cdot {\vec{v}}^{*}\left(t\right)\overset{!}{=}0 \end{array}$$ (22)
- **Proteome density conservation** (Molecular Crowding [16, 19, 22]): The total amount of all enzyme pools summed up together is restricted, which arises from the assumed constant total enzyme concentration *E* ^max^. Therefore, the sum of all relative protein mass is restricted by one,
  $$\begin{array}{c} \sum_{j=1}^{5}\Phi_{j}^{*}=1\;, \end{array}$$
  from which follows, using Eq (20),
  $$\begin{array}{c} \frac{v_{1}^{*}\left(t\right)}{\alpha_{1}^{*}\left(t\right)}+\frac{v_{2}^{*}\left(t\right)}{\alpha_{2}^{*}\left(t\right)}+\frac{v_{3}^{*}\left(t\right)}{\alpha_{3}^{*}\left(t\right)}+\frac{v_{4}^{*}\left(t\right)}{\alpha_{4}^{*}\left(t\right)}+\frac{v_{5}^{*}\left(t\right)}{\alpha_{5}^{*}\left(t\right)}=1\;. \end{array}$$ (23)
  This density conservation constrains the allocation of cellular resources [16, 17, 26]. Our model basically incorporates a three component partition of the proteome [11], namely permeases *ϕ*_1_, *ϕ*_2_, metabolic enzymes *ϕ*_3_, *ϕ*_4_, and ribosomes *ϕ*_5_. The cellular system has to distribute its constrained protein resources between those three components.

### Perception

Perception is the key to proper regulation. Depending on the perceived extracellular nutrient availability, the system’s controller regulates its metabolism differently. We define two kinds of perception, namely the extracellular and intracellular perception. In the case of extracellular perception the cell regulates its metabolism exclusively in response to extracellular nutrient information, while in the case of intracellular perception the opposite holds. In the latter case the cell indirectly recognizes nutrient availability by perceiving intracellular metabolic information.

Looking at Fig 3 one understands why extracellular perception effectively has to act as a feedforward loop while intracellular perception acts as feedback loop on the regulation. Assuming extracellular perception, the information about changes in external nutrient availability have already entered the controller before the cell is able to take them up. Thus, pathways are regulated in response to changes in the environment, even before nutrients enter the metabolism. Contrarily assuming intracellular perception, the information about external nutrients enters the controller not before nutrients have already been transported inside the cell. Thus, pathways are regulated in response to changes in intracellular nutrient concentrations, some time after the nutrient availability has changed in the environment. The cell indirectly perceives its environment and slowly adapts by a feedback control mechanisms.

The incorporation of perception into the above presented mathematical context is done by defining two types of proteome density conservation (see Eq (23)) according to both perception types. Since, intracellular perception is equivalent to an exclusive information about intracellular metabolite pools, only the intracellular quantities *α*_3_, *α*_4_, *α*_5_ enter the conservation equation of a system with intracellular perception.

$$\begin{array}{c} \frac{v_{1}^{*}\left(t\right)}{\alpha_{3}\left(t\right)}+\frac{v_{2}^{*}\left(t\right)}{\alpha_{4}\left(t\right)}+\frac{v_{3}^{*}\left(t\right)}{\alpha_{5}\left(t\right)}+\frac{v_{4}^{*}\left(t\right)}{\alpha_{5}\left(t\right)}+\frac{v_{5}^{*}\left(t\right)}{\alpha_{5}\left(t\right)}=1 \end{array}$$ (24)

Extracellular perception is equivalent to an exclusive information about the extracellular nutrient availability. Therefore, only the extracellular quantities *α*_1_ and *α*_2_ enter the conservation equation of a system with extracellular perception.

$$\begin{array}{c} \frac{v_{1}^{*}\left(t\right)}{\alpha_{1}\left(t\right)}+\frac{v_{2}^{*}\left(t\right)}{\alpha_{2}\left(t\right)}+\frac{v_{3}^{*}\left(t\right)}{\alpha_{1}\left(t\right)}+\frac{v_{4}^{*}\left(t\right)}{\alpha_{2}\left(t\right)}+\frac{v_{5}^{*}\left(t\right)}{\left(\alpha_{1}\left(t\right)+\alpha_{2}\left(t\right)\right)}=1 \end{array}$$ (25)

### Determining the actual value: protein synthesis & metabolism

To determine the actual values of the enzyme and metabolite pools, the metabolic network (Fig 5B) with relative mass fluxes *λ*_*j*_ and *ϕ*_*j*_ has to be used. The actual system can be modeled by a system of 10 coupled ordinary differential equations:

$$\begin{array}{c} \frac{d}{dt}\lambda_{j}\left(t\right)=\alpha_{Y}\left(t\right)\cdot \Phi_{Y}\left(t\right)\;-\;\alpha_{j}\left(t\right)\cdot \Phi_{j}\left(t\right) \end{array}$$ (26)

$$\begin{array}{c} \frac{d}{dt}\Phi_{j}\left(t\right)=v_{\text{growth}}\left(t\right)\cdot [\Phi_{j}^{*}\left(t\right)-\Phi_{j}\left(t\right)]\;, \end{array}$$ (27)

where

$$\begin{array}{c} v_{\text{growth}}\left(t\right)=\alpha_{5}\left(t\right)\cdot \Phi_{5}\left(t\right) \end{array}$$

and

$$\begin{array}{c} \alpha_{j}\left(t\right)=\frac{\lambda_{j}\left(t\right)}{\frac{K_{M}^{\left(j\right)}}{E^{\text{max}}}+\lambda_{j}\left(t\right)}\cdot k_{j}\;. \end{array}$$

Here, the index *Y* denotes the upstream metabolites and enzymes.

### Simulation

To evaluate the fitness benefit due to perception in dependency of environmental fluctuations, a competing species experiment in a fluctuating environment was simulated. While each species exclusively perceives its environment according to intracellular or extracellular perception, the metabolic and regulatory mechanisms are similarly based on the above presented mathematical model. Hence, the only difference between both species is the perception type.

The computer simulation of each species is implemented according to the block diagram Fig 3, which produces the dynamic behavior of growth rate, enzyme and metabolite concentrations, and relative protein synthesis rate (control function). The metabolic network is regulated by a flux balance analysis (FBA) based optimization process [21, 25, 27] (control process), which maximizes cellular growth rate [24] with respect to constant proteome density [19, 20] and optimal enzyme-resource allocation [23]. Particularly, our simulation of the pool dynamics can be regarded as some type of dynamic FBA with quasi-steady-state assumption. This assumption includes discretizing the time into time intervals Δ*t* of constant growth rate *v*_growth_ = const. and regulation (control) *γ*_*j*_ = const., whereas the former is kept constant for the enzyme dynamics only. During an interval Δ*t*, the enzyme and metabolite levels are variable and determined by the system of coupled differential equations, Eqs (26) and (27). At the end of each time step Δ*t* the controller computes the desired enzyme levels *γ*_*j*_ by linear programing within FBA on the basis of the perceived metabolite levels $\lambda_{j}^{*}$ (Eq (24) or Eq (25)). Finally, the updated growth rate *v*_growth_ and regulation *γ*_*j*_ are taken to repeat this procedure for the next time step. The difference of our simulation to conventional dynamic FBA [28, 29] is the notion of a control system, which is rather an element of cybernetic modeling [30].

To obtain regulatory and growth dynamics of the cell which are independent of initial conditions, the simulation operates until both species show a stable periodic behavior. The process of obtaining a stable periodic behavior simulates an evolutionary process in which the cell adapts to an environment with highly predictable fluctuations in nutrient availability. Having attained stability, one periodic growth rate interval is taken to compute the average growth rate, which is the measure for fitness. The whole procedure is repeated for different fluctuation frequencies and therefore yields a frequency dependent plot of the average growth rate. In conclusion, the computer simulation delivers a frequency dependent plot of the species’ fitness as well as the underlying dynamic behavior of metabolism and regulation.

## Results

### Simulation: average growth rate for different switching times

**Fig 6:**
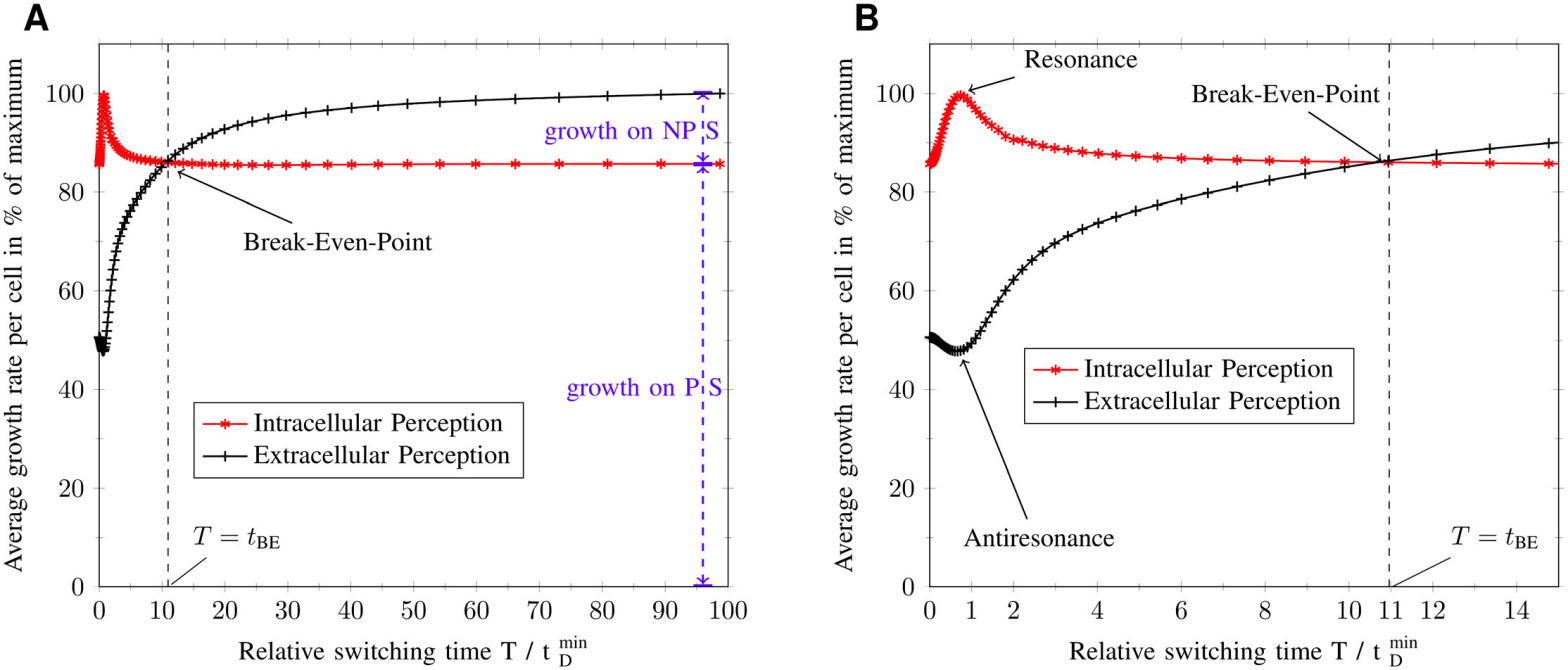
Simulation results of competing species experiment in a fluctuating nutrient environment. Average growth rate for different relative switching times $T/t_{D}^{\text{min}}$ and perception types, whereas $t_{D}^{\text{min}}$ denotes the minimum cellular doubling time. The average growth rate is normalized by its maximal observable value for the sake of generality. The dashed black line at the break-even point *t*_BE_ divides fluctuating environments in regimes of fast *T* = [0, *t*_BE_] and slow *T* =]*t*_BE_, 100] fluctuations. (A) Average growth rate for the interval $T/t_{D}^{\text{min}}=[0,100]$. While the self-replicator with intracellular perception only grows on preferential sugar (PS), the one with extracellular perception also grows on non-preferential sugar (NPS). These contributions to the average growth rate can be seen for the steady state value. (B) Average growth rate for the interval $T/t_{D}^{\text{min}}=[0,15]$.

To determine the frequency regimes in which the intracellular perception is evolutionary more beneficial than the extracellular perception, the average growth rate of the *intracellular perceiving system* (IPS) and *extracellular perceiving system* (EPS) was plotted against the relative switching time $T/t_{D}^{\text{min}}$, as can be seen in Fig 6.

The modeled self-replicators live in a highly predictable ecology, which fluctuates between two environments, namely the non-preferential sugar (NPS) and the preferential sugar (PS) environment. Both sugar types are always present, whereas their concentration fluctuates with respect to the environment. In the NPS environment the NPS possess 50% of the maximum sugar concentration, while the concentration of the PS is as low as 0.25%, which can be regarded as zero. In the PS environment the NPS concentration immediately decreases to 0.25%, while the PS transfers to a maximum concentration of 100%. For preferentiality in vitro, a difference between maximum PS and NPS concentration is not necessary, because changes in fluxes are caused by the quality of the sugar types, i.e the uptake rate. Nevertheless, this model feature guarantees sugar preferentiality in silico without loss of generality. The duration of one environment, PS or NPS, is called the switching time *T*. The reciprocal value of the switching time is exactly the frequency *f*: = 1/*T* of the fluctuations.

To gain a more general view all time quantities are normalized by the minimum cellular response time $t_{R}^{\text{min}}=const.$, which corresponds to the time the cellular system needs to adapt to a constant PS environment. The response time is defined as the time, the cellular system needs to finish 50% of its regulatory work. Specifically it is the average time, the relative enzyme masses $\vec{\phi }$ need to reach half the way between initial $\vec{\phi }\left(t_{0}\right)$ and desired value ${\vec{\phi }}^{*}$.

$$\begin{array}{c} \vec{\phi }\left(t_{R}\right):=\frac{1}{2}\cdot \left({\vec{\phi }}^{*}-\vec{\phi }\left(t_{0}\right)\right) \end{array}$$ (28)

Here, the initial values at time *t*_0_ are the steady state values in the NPS environment. While the minimum response time gives an upper speed limit of cellular adaptation to changing nutrient availability, the cellular doubling time is experimentally more accessible. Assuming no protein degradation, the minimum response time $t_{R}^{\text{min}}$ measures approximately the time a cell needs to double itself once in a constant PS environment, i.e. the minimum cellular doubling time $t_{D}^{\text{min}}=const.$ (minimum generation time) [31]. This minimum doubling time $t_{D}^{\text{min}}$ is constant and corresponds to the maximum growth rate that is achievable. Hence, normalization by the minimum response time can be interpreted as normalization by the minimum cellular doubling time generating the relative switching time $T/t_{D}^{\text{min}}$ and relative time $t/t_{D}^{\text{min}}$. These quantitates produce an organism-independent view on average growth rate and regulatory dynamics, which makes Fig 6 valid for all exponentially growing microorganisms.

Each point in Fig 6 represents the average growth rate for a given relative switching time, that is for a given fluctuation frequency. The average growth rate ${\bar{v}}_{\text{growth}}(T)$ is defined as the time integral over the growth rate dynamics $v_{\text{growth}}^{\left(T\right)}(t)$ divided by one period of fluctuations, specifically twice the switching time.

$$\begin{array}{c} {\bar{v}}_{\text{growth}}\left(T\right):=\frac{1}{2T}\int_{t_{0}}^{t_{0}+2T}v_{\text{growth}}^{\left(T\right)}\left(t\right)\;dt \end{array}$$ (29)

There are four switching time points, which are of interest for a qualitative analysis of the average growth rate. These are (i) *T* approaching zero, (ii) *T* around the minimum response time (minimum doubling time), (iii) *T* at the break-even point *t*_*BE*_, and (iv) *T* approaching infinity. The break-even point divides Fig 6 into two regimes, which are the fast fluctuating regime *T* ∈]0, *t*_*BE*_] and the slowly fluctuating regime *T* ∈ [*t*_*BE*_, ∞[. Inside the first regime the IPS has a larger average growth rate, whereas the EPS grows faster in the second one. For infinitely large switching times, the cells go into steady state. The steady state average growth values can be assigned to contributions due to full adaptation to the PS or NPS environment. As will be seen below, the IPS only adapts to the PS environment, which is equivalent to a cellular system under permanent carbon catabolite repression. Therefore, its steady state value in average growth rate is the contribution ${\bar{v}}_{\text{growth}}^{PS}$ due to exclusive PS adaptation. The EPS adapts fully to both sugar types when in steady state and will only utilize carbon catabolite repression if there are relevant amounts of PS in the environment. Thus, the difference between steady state values of EPS and IPS is exactly the contribution ${\bar{v}}_{\text{growth}}^{NPS}$ caused by adapting completely to NPS surrounding. The contribution to exclusive adaptation to the PS environment has to be larger than the one for the NPS environment, because this is actually the definition of sugar preferentiality. In the here presented environmental example, ${\bar{v}}_{\text{growth}}^{NPS}=15\%$ and ${\bar{v}}_{\text{growth}}^{PS}=85\%$. In conclusion, the intracellular perception, yielding permanent carbon catabolite repression, is evolutionary more beneficial for switching times *T* ∈]0, *t*_*BE*_] and the extracellular perception is more beneficial for *T* ∈ [*t*_*BE*_, ∞[.

### Simulation: actual value

To understand the underlying regulatory principles of the results of Fig 6, the control, enzyme pool, metabolite pool and growth rate dynamics were analyzed at representative relative switching time values. The control dynamics can be understood as the dynamics of the relative protein synthesis rate $\vec{\gamma }\left(t\right)$.

**Mixed environments (*T* → 0)** If the switching time converges towards zero, the cellular system will no longer be able to distinguish between the two environments. Therefore, the cell will perceive a mixed environment. Further, the cell has no time to adapt to any individual environment, since the nutrient fluctuations are much faster than the minimum response time ($T<<t_{R}^{\text{min}}$). There are two regulatory ways to handle this situation, used by the EPS and the IPS, respectively. First, the cell can go into a mixed state, which responds to both environments at the same time. Because of limited resources, according to constant proteome density, the cell adapts partly and gains only half, 50%, of its possible average growth rate (see Fig 6 for $T/t_{D}^{\text{min}}\to 0$). This is the regulatory principle of the EPS. Secondly, the cell can go into and stay in the state of the preferential sugar (PS) environment. This gives rise to no nutrient uptake in the NPS environment and a maximum nutrient uptake in the PS environment. Due to this one-sided adaptation to the PS, the cell gains an average growth rate below the maximum(100%) but higher than 50%. This is the regulatory principle of the IPS.

**Resonance and antiresonance point ($T=\tau \approx t_{R}^{\text{min}}\approx t_{D}^{\text{min}}$)** If the switching time approaches the minimum response time $t_{R}^{\text{min}}$ approximated by the minimum cellular doubling time $t_{D}^{\text{min}}$, the regulatory effects will be observable. By approaching $t_{D}^{\text{min}}$ another quantity becomes relevant, namely the time delay *τ* due to nutrient signaling, which is considered to be approximately equal to $t_{D}^{\text{min}}$. This signaling time delay reflects the adaptation kinetics of the underlying metabolic network and thus is present in both systems, EPS as well as IPS. After changing the relative enzyme masses, it takes this time delay to observe an effect on the growth rate. Thus, any regulatory action will take effect only after *τ*. Moreover, the IPS needs this time to perceive its surrounding, before even being able to take any proper regulatory steps.

**Fig 7:**
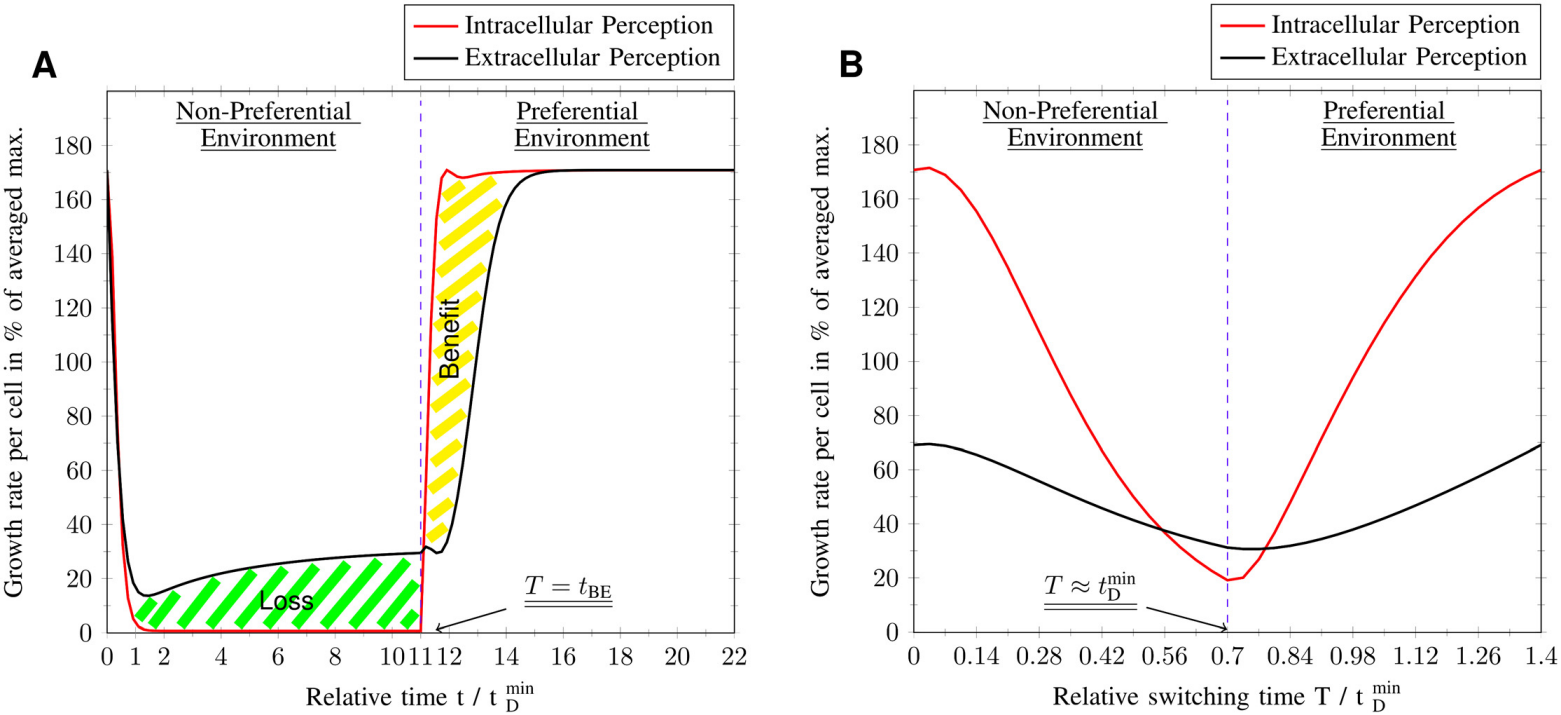
Growth rate dynamics at the break-even point and resonance point. The plot shows one period 2*T* of fluctuations between non-preferential and preferential environment, whereas the dashed black line separates both environments (periodic boundary conditions). Time *t* is normalized by the minimum cellular doubling time $t_{D}^{\text{min}}$. (A) Growth benefit and loss of intracellular perception due to exclusive adaptation to preferential sugar. The area between both graphs is the measure for benefit and cost relative to both perception types. (B) Growth dynamics at the resonance point $T/t_{D}^{\text{min}}=0.7\approx 1$. The large amplitude of the growth rate fluctuations for intracellular perception leads to an optimal average performance and is caused by the resonance of cellular response time with switching time *T* between environments.

**Fig 8:**
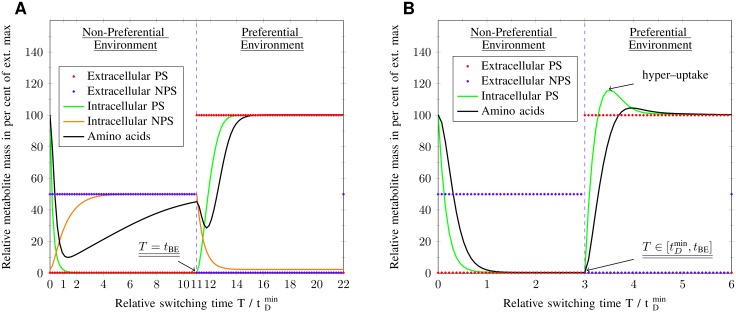
Metabolite pool dynamics. The plot shows one period 2*T* of fluctuations between non-preferential and preferential environment, whereas the dashed black line separates both environments (periodic boundary conditions). Time *t* is normalized by the minimum cellular doubling time $t_{D}^{\text{min}}$. (A) Extracellular perception at break-even point: both sugar types, preferential (PS) and non-preferential (NPS), are taken up. The condition of constant metabolite pools, caused by optimal enzymatic resource allocation, is approached for switching times *T* larger than the break-even point *t*_BE_. (B) Intracellular perception at $T/t_{D}^{\text{min}}=3$ between resonance point and break-even point: only PS is taken up with an increased PS uptake during the PS environment, which is the cause for the optimal growth at the resonance point.

While the EPS perceives its nutrient environment in an exact and instantaneous manner, the IPS has a limited and delayed vision of its surrounding (see Fig 7A, where the IPS does not grow at all in the NPS environment and Fig 8A, where there is no NPS uptake at all). There are two main features that distinguish the IPS from the EPS or the intracellular perception from the extracellular perception, respectively. First, the IPS has to wait for a time delay *τ* until the nutrient signaling affects the intracellular metabolic pools, in order to sense what has happened externally. Secondly, the IPS deactivates the NPS pathway, which prevents the system to perceive NPS. Hence, the IPS is not able to sense the switching between environments with $T\leq t_{D}^{\text{min}}$. Based on these perception types, the EPS adapts to each individual environment whereas the IPS adapts to the one with PS, only. Additionally, the IPS prepares itself for an increased PS uptake by hyper-up-regulation of PS uptake transporters during the NPS environment. This increased PS uptake only occurs for a short time interval (see Fig 8B), so that an environmental change with a switching time similar to the signaling time delay produces a resonance effect (see Fig 7B).

Considering a switching time that equals the signaling time delay $T=\tau \approx t_{D}^{\text{min}}$, the EPS yields an antiresonance effect with the wrong pathway regulation at the wrong time. This effect generates the worst average growth rate possible (< 50%), whereas the growth is even smaller than for adapting to both sugar types simultaneously in a mixed environment. In contrary, the IPS supplies the perfect regulation with the best possible result (100%), resulting from a resonance effect. Concluding, if there is no time for regulation to act on growth rate, it will be beneficial to focus on PS and use the PS gap phase to prepare for the PS environment.

**Break-even point (*T* = *t*_*BE*_)** If the switching time *T* approaches the break-even point *t*_*BE*_, the EPS will approach the IPS in average growth rate. Specifically, the Growth Benefit, due to adaptation to the NPS environment, will exceed its associated growth loss.

The IPS perceives correctly the extracellular PS concentration in both environments and imposes an constant activation of the PS pathway. On the other hand, the EPS alternately activates and deactivates NPS and PS pathways to adapt to the environment. There is enough time for full response and the EPS can implement the control function (desired value) into reality. Nevertheless, the IPS stays fixed inside the state of hyper-up-regulation throughout the whole NPS environment. After its amino acid pool becomes zero, there is no driving growth rate to regulate pathways.

To understand, why EPS and IPS approach the same average growth rate at the break-even point one has to understand the concept of growth benefit and growth loss due to the underlying regulatory strategy. Simply spoken, the IPS only uses the PS pathway to grow, while the EPS uses both pathways. To decide which of the strategies is more favorable, one has to explain for which cases using two pathways is more favorable than only one. The advantage of the IPS is that it does not need to adapt to the PS, so it gains a maximal growth rate while the EPS still is adapting to the new environment. This represents a growth benefit for the IPS (see Fig 7). The advantage of the EPS is that it can also grow in the NPS while the IPS goes into a type of growth arrest. This represents a growth loss for the IPS (see Fig 7). Concluding, these two growth effect are exactly equal at the break-even point.

**Steady state (*T* → ∞) & limits of the model** If the switching time becomes larger than the break-even point and approaches infinity, the extracellular perception and therefore the EPS will have the dominant strategy. The cells enter steady state, therefore balanced fluxes, optimal resource allocation and constant metabolite pools are realized. The latter feature can be seen in Fig 8, where the metabolite pool concentrations converge to the one of extracellular nutrients.

A switching time that lasts an infinitely long time is the equivalent of an nutrient environment that stays constant and does not fluctuate at all. On one hand, the EPS imposes full adaptation to the respective nutrient environments, which intuitively makes sense for an infinitely large switching time. On the other hand, the IPS only adapts to the PS and thus resides in growth arrest during NPS surrounding (Fig 7A). The IPS traps itself in using PS until this resource is exhausted. More reasonable, the resulting drop in growth rate should promote the transition to stringent response, right after the break-even point *t*_*BE*_. Stringent response would enable the IPS to activate the NPS pathway by bypassing the limited nutrient perception. Then, the average growth rate of IPS would probably converge to the one of the EPS.

Since our research questions is asking for regulatory principles in fluctuating environments, the case of infinitely large switching time is not relevant. It is only necessary to understand the limits of this model. After the break-even point *t*_*BE*_, the model system makes no valid predictions for the IPS. It has no stringent response and thus can theoretically grow on the smallest amount of PS, which is 0.25% in the here presented example. This is a physiological unrealistic case. In order to add stringent response to the model, a constraint on the minimal detectable nutrient concentration could be introduced. If the PS concentration goes below this constraint, stringent response will be turned on.

## Discussion

This study indicates that indirect intracellular perception of extracellular nutrient availability can give rise to a growth benefit under situations where the up and down regulation of pathways cannot follow the fast changes of the nutrient environment. Although intracellular perception carries less information about the actual environmental conditions, this regulatory mechanism enables exponentially growing organisms to gain maximal average growth if nutrient concentrations fluctuate on timescales comparable to the minimum generation time.

In our simulation, a system with intracellular perception responds to strong fluctuations by keeping preferential nutrient pathways activated and non-preferential pathways inactivated. As a result the cell can take up preferential nutrients as soon as they are available without any prior regulation. This regulatory strategy is a good example for *minimal adjustment*. According to Schuetz et al. [1] there is a trade-off between *optimality* under one given condition and *minimal adjustment* between different conditions, i.e. Pareto optimality [32]. In other words, cells will tune metabolic pathways to obtain optimal growth if surrounded by a constant environment. Contrarily, in a fluctuating environment, cells will regulate their pathways to respond to environmental changes by minimal adjustment of pathways. In this sense, intracellular perception gives rise to a regulation of *minimal adjustment*, which is dominant under fast environmental changes. Additionally, our results show that the notion of optimality is also given under fluctuating conditions, since minimal adjustment is a consequence of maximizing an objective function averaged over the range of conditions.

Moreover, our model of intracellular perception is in agreement with the phenomenon of carbon catabolite repression [7, 9], if cells are not able to distinguish between different conditions anymore, i.e. the fluctuation frequency approaches infinity. This situation is equivalent to a mixed constant environment. While carbon catabolite repression reflects the cell’s affinity to preferential sugars in a stable mixed nutritional surrounding, our results indicate that this mechanism holds under fast fluctuations (around the minimum generation time) as well. To our knowledge, CCR has not been obtained from an mathematical optimization process, before.

Furthermore, our simulation of the growth dynamics produced a break-even point, where the average growth rate of the IPS and EPS are equal (Fig 6). At this point the growth benefit of the IPS in the preferential environment matches the growth loss in the non-preferential environment. Growth benefit and loss arise from the exclusive adaptation to the PS environment (Fig 7A). This is in agreement with the experimental work of Mitchell et al. [33], who have observed anticipation of environmental changes in the sugar metabolism of *E.coli* and *S.cerevisiae*. Mitchell et al. classified the regulatory response to environmental changes into direct and anticipatory regulation, whereas the former regulates its metabolism in direct response and the latter in advanced preparation. Further, they state that an anticipatory response will be evolutionary beneficial if “the benefit gained from anticipation exceeds the cost of early preparation”. We can identify the anticipatory regulation with the IPS and the direct regulation with the EPS. As we have shown intracellular perception yields a preparation for the PS environment during the NPS environment, which can be regarded as an anticipatory behavior. Especially, the hyper-up-regulation of the PS uptake transporter in the presence of NPS environment, which results in the resonance peak of the average growth rate, serves as a good example for anticipatory regulation. This course of action is only beneficial for fluctuating environments with frequency smaller than the break-even frequency. Thus, anticipatory behavior in a highly predictable fluctuating environment can be understood by limited and delayed intracellular perception.

Using our phenomenological computer model, we further showed that extracellular perception is of selective advantage under slow environmental fluctuations. However, it is reasonable to assume that intracellular perception always contributes to some extent to growth control. This hypothesis is supported by the observations of New et al. [34], who have shown that wild *S. cerevisiae* strains divide into sub-populations of specialist and generalists according to their growth rate related response time (lag phase). Generalist will adapt faster to a new carbon environment than specialists if the environment changes from a preferential to a non-preferential carbon source. Our results in Fig 7A for the non-preferential regime exhibit the same relation between growth regulation by means of extracellular perception (EPS) and intracellular perception (IPS). The EPS, like the generalists, adapts faster to the non-preferential environment than the IPS. In this context generalist could be seen as microbes whose growth control mainly depends on extracellular perception, while the contribution of intracellular perception has an bigger impact on the specialist’s growth control. Although, both perception types can be utilized by microorganisms, their contribution to growth control can be differently depending on the individual evolutionary background.

Regarding the IPS, an interesting result of our simulation is the existence of a resonance peak for fluctuations around the minimum generation time. At this peak, the time delay in nutrient perception equals the switching time between environments resulting in optimal fitness. The data-based mathematical model of Mitchell and Pilpel [35] supports our finding as their cellular system shows a fitness peak around 1–2.5 generation times.

To summarize, our work indicates that intracellular perception is of selective advantage and gives rise to CCR in oscillating environments, so that microbes specialize on the preferential nutrient and anticipate it in its absence. In general, intracellular perception could be a fundamental regulatory principle of minimal adjustment to changing conditions. Although our study is limited to a purely qualitative conclusion, due to the simplicity of our approach, the presented model is sufficient to gain insight in the fundamental differences of microbial growth control. In following projects, it would be worthwhile to test our simulation with real metabolic networks, like from the model organisms *E.coli* or *S.cerevisiae*. Moreover, experimental evidence, i.e. competing species experiments, is needed to confirm our theory of the dominance of intracellular perception under fast fluctuations.
